# Critical role of glycosylation in determining the length and structure of T cell epitopes

**DOI:** 10.1186/1745-7580-5-4

**Published:** 2009-09-24

**Authors:** Tamás G Szabó, Robin Palotai, Péter Antal, Itay Tokatly, László Tóthfalusi, Ole Lund, György Nagy, András Falus, Edit I Buzás

**Affiliations:** 1 Department of Genetics, Cell- and Immunobiology, Semmelweis University, Nagyvárad tér 4, Budapest, Hungary; 2 Department of Medical Chemistry, Molecular Biology and Pathobiochemistry, Semmelweis University, Budapest, 1094 Budapest, Tűzoltó u 37-47, Hungary; 3 Department of Measurement and Information Systems, Budapest University of Technology and Economics, Budapest, Pf 91, 1521 Budapest, Hungary; 4 Department of Pharmacodynamics, Semmelweis University, Nagyvárad tér 4, 1089 Budapest, Hungary; 5 Center for Biological Sequence Analysis, Department of Systems Biology, Technical University of Denmark, Building 208, DK-2800 Kongens Lyngby, Denmark; 6 Department of Rheumatology, Semmelweis University, Árpád fejedelem u 7, 1022 Budapest, Hungary; 7 Research Group for Inflammation Biology and Immunogenomics, Hungarian Academy of Sciences, Nagyvárad tér 4, 1089 Budapest, Hungary

## Abstract

**Background:**

Using a combined *in silico *approach, we investigated the glycosylation of T cell epitopes and autoantigens. The present systems biology analysis was made possible by currently available databases (representing full proteomes, known human T cell epitopes and autoantigens) as well as glycosylation prediction tools.

**Results:**

We analyzed the probable glycosylation of human T cell epitope sequences extracted from the ImmuneEpitope Database. Our analysis suggests that in contrast to full length SwissProt entries, only a minimal portion of experimentally verified T cell epitopes is potentially N- or O-glycosylated (2.26% and 1.22%, respectively). Bayesian analysis of entries extracted from the Autoantigen Database suggests a correlation between N-glycosylation and autoantigenicity. The analysis of random generated sequences shows that glycosylation probability is also affected by peptide length. Our data suggest that the lack of peptide glycosylation, a feature that probably favors effective recognition by T cells, might have resulted in a selective advantage for short peptides to become T cell epitopes. The length of T cell epitopes is at the intersection of curves determining specificity and glycosylation probability. Thus, the range of length of naturally occurring T cell epitopes may ensure the maximum specificity with the minimal glycosylation probability.

**Conclusion:**

The findings of this bioinformatical approach shed light on fundamental factors that might have shaped adaptive immunity during evolution. Our data suggest that amino acid sequence-based hypo/non-glycosylation of certain segments of proteins might be substantial for determining T cell immunity/autoimmunity.

## Background

Antigen recognition by the immune system is decisive in the fight against pathogens and tumors. T lymphocytes recognize short, linear peptide fragments (epitopes) of antigenic proteins in context with self MHC.

Posttranslational protein modifications (PTMs) generate an enormous variety of modified protein-derived epitopes. However, the significance of the posttranslationally modified antigenic determinants in shaping the receptor repertoire and determining the outcome of an immune response, is poorly understood. Within the category of PTMs, glycosylation is the most frequent one, and N-glycosylation is the most abundant form of glycosylation in humans. The relative backlog in the understanding of glycoimmunology (as compared to anti-protein immunology) might be explained, at least partially, by the substantial technical difficulties of carbohydrate synthesis and sequencing. Fortunately, in the past years there has been an explosion of progress in glycobiology and also in glycoimmunology. However, despite the extensive work, fundamental questions (such as the role of epitope glycosylation in T cell recognition) remain to be answered.

Glycosylation of a peptide increases its space-filling capacity, thus, altering its interactive surface with the TCR or with MHC. While it is obvious that glycosylation creates new ligands for different B cell receptors, it is yet unclear how it affects epitope recognition by T cells. The aim of this study was to investigate the impact of glycosylation on T cell recognition. According to our hypothesis, glycosylated peptides are less likely to be recognized by T cells because of the glycan moiety. Indeed, there are sporadic experimental data suggesting that peptide glycosylation may interfere with epitope recognition through TCR [1-3]. On the other hand, alterations in protein glycosylation may also play a role also in the pathogenesis of autoimmune diseases. The molecular mimicry theory predicts that a cross-reactive immune response to similar or identical antigen determinants of microbial and human origin, may result in a pathological immune response directed against self antigens. In line with this concept, self-nonself discrimination of exact sequence matches is possible as long as the peptides are differentially glycosylated. Removal of the glycan moiety in pathological conditions might, however, result in a facilitated recognition of a formerly tolerated epitope. In order to test this possibility, we compared the glycosylation frequency of the complete human proteome with that of the experimentally verified T cell epitopes and the bacterial proteins with mimicry potential.

The relevance of epitope glycosylation in the development of autoimmunity has been recently suggested by the finding that an increased substrate flux through N-glycosylation machinery has a therapeutic effect in some autoimmune diseases [4].

Publicly available T cell epitope prediction approaches focus on binding of a given peptide to HLA and not to TCR. Furthermore, it has also been described recently that the currently available *in silico *tools are rather limited in their prediction efficiency, which also differs from allele to allele [5]. This study was motivated by the urge to explore further antigenicity-related characteristics of peptides in order to improve the accuracy of T cell epitope prediction. In order to study the role of epitope glycosylation on T cell immunity, we used combined state of the art bioinformatics tools (databases and neural network-based glycosylation prediction algorithms).

## Materials and methods

### Protein and peptide datasets

Protein sequences analyzed in the study derive from the taxonomic divisions provided by the UniProt KnowledgeBase [6,7]. These data sets contain all entries of UniProtKB/SwissProt database corresponding to a given taxonomic group. In the current study, 16 440 human and 140 337 bacterial entries of the database's taxonomic divisions, all protein sequences available on Aug 22/2007, were used. These sequences were used for prediction of glycosylation and MHC-binding sites, while annotation in the FT (function) line was applied to verify prediction results and also was used for Bayesian statistics. Data conversion from the FASTA format into our database was manually revised, and in very few cases, truncated sequences and annotations to non-appropriate category columns were corrected according to re-retrieved data from SwissProt database.

Experimentally verified human protein-derived T cell epitopes (1782 different entries) were downloaded from IEDB v 1.0 [8,9]. First a transitional database was generated by performing an advanced query for positive T cell response to "structure/chemical type: peptide/protein" and "source species: homo sapiens". The output did not provide any data concerning the MHC I or MHC II restriction of T cell recognition of the given epitope. Given the known length range of MHC I and MHC II binding epitope sequences [10], we considered ≤ 10 and ≥ 13 aa long peptides as MHC I-restricted and MHC II-restricted T cell epitopes, respectively. Any redundancy in the database was eliminated based on the information provided on the source protein of the epitope. The corresponding SwissProt entry was looked up for each source, and amino acid positions within the full length proteins were assessed. A small proportion of retrieved entries that could not be matched with any human SwissProt protein, were excluded from further analysis. Only the unique "epitope sequence-protein of origin-amino acid position" data were used for further analysis. Protein sequences of known autoantigens are listed in the Autoantigen Database [11,12]; we used the whole database, instead of querying particular diseases. Both the sequence and SwissProt ID found here were used to determine autoantigenic (n = 440) and non-autoantigenic (n = 16 000) proteins.

### Predictions

Glycosylation of proteins was assessed by the prediction servers of the Center for Biological Sequence Analysis [13] using the NetOglyc (sensitivity: 76%; specificity 93%) [14,15] and NetNGlyc (sensitivity: 86%; specificity: 61%) [16] servers, trained on a relative large set of experimental data. Other glycosylation prediction methods have been also described [17-21]. From among the currently available glycosylation-related bioinformatics tools summarized recently by Petrescu et al. [22], we used the *in silico *predictions of glycosylation.

N-glycosylation of proteins occurs at a well defined sequence motif (NXS/T), where X can be any amino acid other than proline. This motif is referred to as "conditional" N-glycosylation site throughout the study. Depending on the second amino acid of the triplet, the probability of glycosylation may vary. This probability can be assessed by the prediction servers. N-glycosylation sites predicted as "likely to be glycosylated", are referred to as "probable N-glycosylation sites" in the text.

As N- and O-glycosylation occur in the ER and Golgi, only proteins with a signal peptide (SigP) are expected to be glycosylated. Glycosylation of proteins by other mechanisms (e.g. by Yin-Yang glycosylation) has not been studied here. Besides prediction of SigPs, prediction of the transmembrane regions of proteins was also carried out by servers provided at the CBS homepage [23-27]. The latter predictions were also important because only extracellular domains of proteins are expected to be N-glycosylated. Thus, cytoplasmic and transmembrane sequence regions, predicted to be glycosylated, were ignored. SigPs were predicted by two methods (Artificial Neural Networks and Hidden Markov Model), and in those few cases, in which the results were contradictory, the annotation of SwissProt was accepted. If no annotation suggested the presence of either a SigP or the localization in the ER or Golgi, the protein was considered as one without a SigP. Another CBS prediction server, NetMHCII [28,29] was used to assess potential MHC II ligands for the most common HLA DR alleles [30]. This software was trained on experimental MHC binding assay data in the IEDB and the AntiJen database. The AUC values representing the predictive performance of the method vary between 0.664 and 0.818, depending on the different alleles as described earlier [28]. The prediction yielded an MHC-binding score for each possible 15 amino acid long peptide derived from that protein, and for the sequence of the 9 core amino acids. Both scores were used in our analysis.

### Python Programming Language (Large scale data handling)

In this study, we used Python Programming Language [31]. This script language is becoming increasingly popular in biological research since the availability of BioPython toolkit [32]. It was used for submission of sequences for prediction, for analysis of prediction results, for comparison of sequences and for handling data throughout the study.

The script language was used to detect matching sequences between human and bacterial proteins. It was carried out by searching for ≥ 7 aa long sequence fragments of human proteins in the sequences of bacterial proteins. Python was also used for random selection of 4000 bacterial proteins in order to estimate the correlation between peptide lengths and the number of bacterial-human sequence matches of 4-6 aa length.

Another application of the programming language was to generate amino acid composition-matched random control sequences or to select length-matched peptides from SwissProt protein sequences. Python uses the Mersenne Twister algorithm; a pseudo-random number generator widely used in stochastic applications, like in Monte Carlo simulation [33].

Random control sequences for autoantigens were generated by random sampling of amino acids, giving the same weight to each amino acid as its frequency in the original protein. For random generated T cell epitope sequences, the amino acid weight was determined by assessing the amino acid frequencies among all human proteins with a signal peptide. The weights were as follows: A: 0.070; C: 0.025; D: 0.048; E: 0.067; F: 0.039; G: 0.069; H: 0.026; I: 0.043; K: 0.055; L: 0.099; M: 0.022; N: 0.036; P: 0.063; Q: 0.046; R: 0.057; S: 0.081; T: 0.053; V: 0.062; W: 0.013; Y: 0.029.

### Statistics

Analysis of data was carried out by classical statistical tests (Statistica software package). We also performed a Bayesian analysis of multivariate relevance and interactions using Bayesian networks [34-37]. The discretization of the continuous variables was determined by a priori biological expertise and univariate statistical analysis. Discretization criteria were as follows: sequence length: length (0-500), length (500-100), length (1000<); sequence type (autoantigen or not): seq_type_id (0), seq_type_id (1); presence of signal peptide: signalp (0), signalp (1); presence of Ca binding domain: ca_bind (0), ca_bind (0<); presence of coiled regions: coiled (0), coiled (0<); presence of DNA binding motif: dna_bind (0), dna_bind (0<); presence of helical structure: helix (0<), helix (0); presence of metal binding region: metal (0), metal (0<); nucleoprotein binding: np_bind (0), np_bind (0<); presence of strands: strand (0), strand (1-10), strand (10<); presence of turns: turn (0), turn (0<); presence of zinc finger motif: zn_fing (0), zn_fing (0<); presence of O-glycosylation site between two disulphide bonds: o_disulphide (0), o_disulphide (1), o_disulphide (1<); O-glycosylation site coinciding with a repeat sequence: o_repeat (0), o_repeat (1-10), o_repeat (10<); predicted intracellular O-glycosylation site: o_IC (0), o_IC (1-10), o_IC (10<); extracellular O-glycosylation site: o_EC (0), o_EC (1-10), o_EC (10<); O- glycosylation site within mitochondrial proteins: o_mitochondrial (0), o_mitochondrial (0<); O-glycosylation site within a transmembrane domain: o_transmembr (0), o_transmembr (1); N-glycosylation site between two disulphide bonds: n_disulphide (0), n_disulphide (1), n_disulphide (1<); N-glycosylation site coinciding with a repeat sequence n_repeat (0), n_repeat (1), n_repeat (2), n_repeat (3); predicted intracellular N-glycosylation site: n_IC (0), n_IC (1), n_IC (2); extracellular O-glycosylation site: n_EC (0), n_EC (1), n_EC (1<), N-glycosylation site within mitochondrial proteins: n_mitochondrial (0), n_mitochondrial (0<); N-glycosylation site within a transmembrane domain: n_transmembr (0), n_transmembr (0<); number of disulphide bridges: sum_disulphide (0), sum_disulphide (1-10), sum_disulphide (10<); number of repeats: sum_repeat (0), sum_repeat (1-10), sum_repeat (10<); number of a transmembrane domains: sum_transmembr (0), sum_transmembr (1-4), sum_transmembr (4<); distance of N-glycosylation sites: n_distance (<0.65), n_distance (0.65-0.85), n_distance (0.85<); distance of O-glycosylation sites: o_distance (<0.35), o_distance (0.35-0.8), o_distance (0.8<); number of N-glycosylation sites: num_nglyc (0), num_nglyc (1), num_nglyc (2), num_nglyc (3-4), num_nglyc (5<); number of N-glycosylation sites with more than 75% probability of N-glycosylation site occupancy: num_nglyc3 (0), num_nglyc3 (1-2), num_nglyc3 (2<); number of O-glycosylation sites: num_oglyc (0), num_oglyc (1-10), num_oglyc (10<). The length of the burn-in was selected using Geweke's z-score test and the R value of the multiple-chain method of Gelman-Rubin [35,37]. The length of the MCMC simulation was selected to decrease the variances of the MCMC estimates below 0.01.

## Results

### Glycosylation of the human proteome

In order to generate a basis for comparison with antigens and antigen-derived T cell epitopes, we set to assess the glycosylation of the human proteome based on sequence analysis of entries extracted from SwissProt/UniProtKB. To determine if amino acids that are implicated in glycosylation (e.g. Asn, Ser, Thr), are differentially represented among the 20 amino acids in the human proteome, we analyzed 16 440 human protein sequences. We found that serine represented 8.07% of all amino acids in humans, while threonine was found at 5.27% and asparagine at 3.6% frequency.

Since glycosylation is expected to imply to SigP-containing proteins only, we identified SigP-containing sequences in t SwissProt/UniProtKB database. This filtering yielded in 24.92% of all human proteins. No significant difference was identified in the amino acid composition of proteins with a SigP (7.74% serine, 5.72% threonine and 3.85% asparagine) in comparison with the whole proteome.

The next important step was to compare the length of the members of the proteome with that of the SigP-bearing proteins. The protein length of the human proteome was 543 amino acids ± 4 (mean ± standard error). Proteins with a SigP did not differ significantly from this; they were characterized by a length of 522 ± 8 amino acids (mean ± standard error).

We next focused on the potential glycosylation of proteins with and without SigP. We found that 73.54% of the SigP peptide-bearing sequences were predicted to carry N-glycosylation, while approximately 55.43% of these proteins were predicted to be O-glycosylated. Considering SigP as a prerequisite for both N- and O-glycosylations, only 18.31% of the whole human proteome was found to be N-glycosylated and 13.82% O-glycosylated.

**Figure 1:**
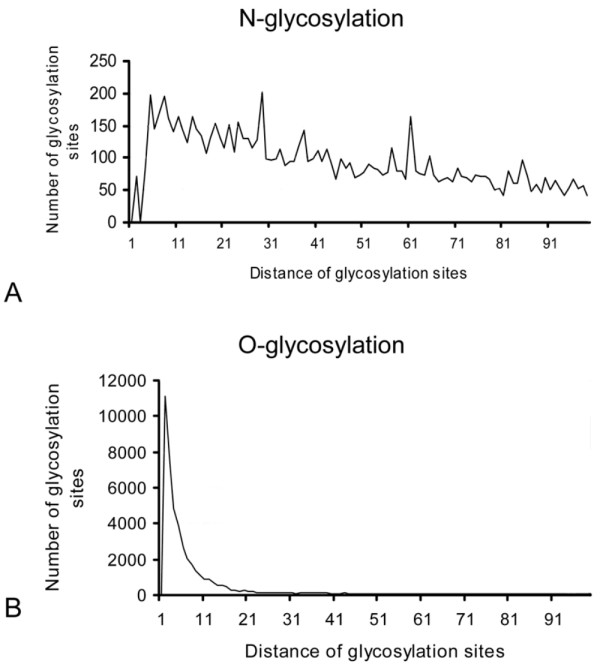
**Average distances of glycosylation sites**. The histogram shows frequency distribution of distances between glycosylation sites. It shows a markedly different pattern of N- and O-glycosylation. Most probable O-glycosylation sites are found less than 10 amino acids far from each other. In contrast, distances between probable N-glycosylation sites are highly variable. This indicates that O-glycosylation sites are found in clusters, while conditional N-glycosylation sites are more equally distributed throughout a protein sequence.

We also compared the distribution of glycosylation sites along the proteins. As expected, the pattern of N- and O-glycosylation of proteins showed a marked difference: while N-glycosylation sites were relatively distant from one another, O-glycosylation occurred in clusters (Figures 1A and 1B).

### Glycosylation of human T cell epitopes

To analyze the probable glycosylation of T cell epitopes, we extracted all linear human protein-derived T cell epitope sequences from the ImmuneEpitope Database (IEDB). In this first analysis, we did not investigate MHC I and MHC II-related epitopes separately, since the basic principles of the trimolecular complex formation by a TCR and a peptide in context with self MHC, are highly similar. Since the amino acid environment of a possible glycosylation site also carries important information for the prediction algorithm to assess the probability of glycosylation, predictions were not carried out directly on the experimentally verified T cell epitope peptide sequences. Instead, the glycosylation was predicted for the full length protein from which the given T cell epitope was derived, and the information for the T cell epitope sequences, found in the IEDB, was deduced from these data.

We found that only a very small portion of all experimentally verified human T cell epitopes were potentially N- or O-glycosylated. Only 2.26% of all T cell epitopes were predicted to be N-glycosylated, and 1.22% were predicted to be O-glycosylated. When considering the SigP-bearing protein-derived epitopes only, 5.68% of the experimentally verified T cell epitopes, and from among them 5.56% of MHC II-restricted epitopes were N-glycosylated, while O-glycosylation characterized 3.06% and 3.61% of the sequences, respectively. We could not identify any amino acid position N- or C-terminally from the experimentally verified T cell epitopes that was more prone to either N- or O-glycosylation.

Although it may seem trivial that longer sequences are more likely to contain more glycosylation motifs, any evolutionary pressure affecting the glycosylation of epitopes could significantly bias this correlation. To test this possibility, we generated randomly 100 different n, 5n and 25n long sequences (where n is the number of amino acids of the original epitope) as controls for each verified T cell epitope in the IEDB. We found that 22.07% of the 5n, and 70.54% of the 25n long sequences contained the NXS/T sequence (not shown). This 70.54% is very close to the portion found in SwissProt human entries. By generating 10000 random sequences for each peptide length from 7-30 amino acids, a linear correlation could be found between the peptide length and the presence of NXS/T motif as a possible N-glycosylation site (r = 0.99).

As an alternative approach, we also analyzed the probability of glycosylation as a function of length, and received concordant results (data not shown). Thus, the reduced glycosylation rate of T cell epitopes could theoretically result from the shortness of these sequences in comparison with full-length proteins.

To test if sequence length was the only factor effecting glycosylation, matched random control sequences were also tested. As a first approach, we randomly generated pools of 100 different, composition-matched amino acid sequences of the same length for each T cell epitope in the IEDB. Only 4.18% of all randomly generated sequences and 7.73% of randomly generated controls for MHC II restricted epitopes contained the NXS/T N-glycosylation motif, which did not differ significantly from the rates found analyzing all T cell epitopes (3.9%) or MHC II- restricted epitope sequences (4.72%) (chi square, p = 0.21).

**Figure 2:**
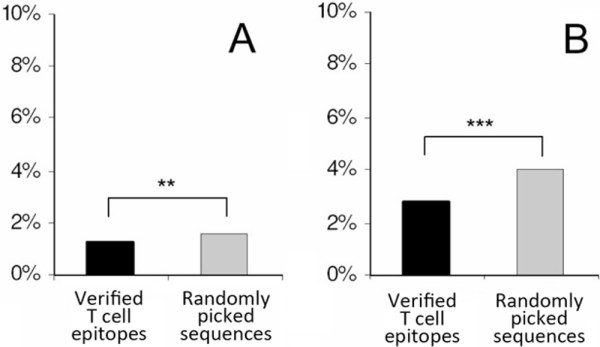
**The probability of N- and O-glycosylation**. Experimentally verified T cell epitopes (filled columns) (found in ImmuneEpitope Database) and randomly selected peptides with identical length from the same protein (gray columns) were analyzed for probable N- and O-glycosylation using Artificial Neural Network-based predictions. Probable glycosylation of ≥ 9 amino acid long peptides is shown in the figure. A peptide was considered to have probable glycosylation if the position of the predicted glycosylation site within the protein coincided with the position of the peptide. Figure 2A shows comparison of the numbers of predicted O-glycosylation sites for each experimentally verified T cell epitope and a randomly selected peptide with identical length from the same protein (Wilcoxon, p = 0.0019). Figure 2B shows comparison of predicted N-glycosylation of randomly selected, length-matched sequences and the functional T cell epitopes (Wilcoxon test, p = 0.00006).

As another approach of control group design, for each experimentally verified T cell epitope, we randomly selected a peptide control of identical length from the very same protein antigen. The experimentally verified epitopes (n = 1722) and the randomly chosen matched sequences derived from the same antigen as the T cell epitope (n = 1722), were compared for the presence of the NXS/T motif. From among all verified T cell epitopes, 75 contained the NXS/T motif, while it was present in 115 of the randomly selected matched sequences. To test its significance, we used the Wilcoxon signed-rank test in which each control peptide was paired with an epitope derived from the same protein (p = 0.005). Probably due to the smaller sample size, the difference was significant only when MHC II-restricted T cell epitopes were considered (Wilcoxon p = 0.056). After testing the presence of the NXS/T, we also used neural network-based glycosylation predictions to compare the number of probable N-glycosylation sites for each experimentally verified T cell epitope and a randomly selected peptide pair of it with identical length from the same protein. We found that 1.437 times more randomly picked sequences of the same protein origin were predicted to carry N-glycosylation in comparison with the functional T cell epitopes (Figure 2, Wilcoxon p = 0.00006). No significant difference was found, however, when we compared MHC I- or II-restricted peptides with the corresponding random controls (1.333 and 1.519 times more sequences were predicted to be N-glycosylated than the controls, respectively). This could have, again, resulted from the smaller sample sizes.

As there is no consensus sequence motif of O-glycosylation sites (Ser and Thr being highly abundant in proteins), only a prediction algorithm was used to assess the probable O-glycosylation. Experimentally verified T cell epitopes were not significantly different from randomly picked controls regarding their O-glycosylation, except for MHC I-restricted epitopes, in which the number of O-glycosylation sites was only 42.1% of the randomly picked control pairs (Wilcoxon p = 0.0257).

### Predicted MHC II-binding peptides

Since most SigP-possessing human proteins fall into the category of either secreted or plasma membrane molecules, they are likely to be presented by MHC II molecules (similarly to bacterial proteins). To determine peptide sequences with high probability to bind to some of the most common human MHC II alleles, we analyzed all human proteins possessing SigP. Using the most sensitive prediction method available [28], we studied the MHC II binding of N- and O-glycosylated peptides.

**Figure 3:**
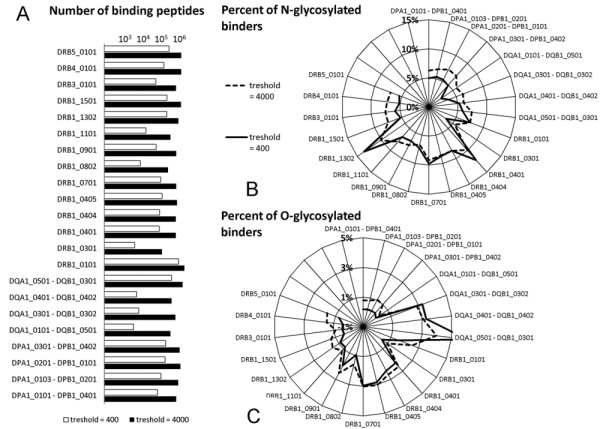
**Effect of glycosylation on MHC binding**. Binding of peptides derived from SwissProt/UniProtKB entries with SigP to the most common human HLA II alleles was predicted by NetMHCII software. **A**. HLA alleles showed significant differences in the numbers of predicted binding peptides. **B**. Proportion of peptides predicted to carry an N-glycosylation site is very different among different MHC II molecules. Most HLA DR alleles are found to be more prone tolerate N-glycosylation, than HLA DP and DQ. **C**. The frequency of probable O-glycosylation among peptides binding to different HLA alleles is shown on a polar graph.

HLA alleles showed significant differences in the numbers of predicted strong and weak binder peptides derived from SigP-containing human proteins. The software uses binding scores 50 and 500 as threshold values for strong or weak peptide binding, respectively. However, a recent study shows that its performance can be enhanced by shifting these values to 400 and 4000 [38]. Similarly to what has been seen at 50 and 500 scores, at 400 and 4000 threshold values we could observe differential peptide binding by different HLA alleles (Figure 3A). HLA DRB1* 0101 showed the most promiscuous peptide binding (Figure 3A), while HLA alleles HLA DRB1*0301, DRB1*0802 and DRB3*0101 were predicted to bind a significantly reduced proportion of all human peptides derived from proteins with SigP (Figure 3A). Similarly, HLA DQA1*0501 - DQB1*0301 showed significantly higher promiscuity as compared to other HLA DQ alleles. This differential permissivity to bind peptide ligands did not show a correlation with the published area under the curve (AUC) values that reflect the predictive performances of the software for the individual MHC alleles [28].

To further address the question of MHC II binding, we next examined the glycosylation of peptides predicted to bind to the MHC II alleles, mainly HLA DR1 (eg. HLA DRB1*0401 and HLA DRB1* 1302). Most HLA DP and DQ alleles seem to tolerate N-glycosylation much less (Figure 3B) than HLA DR, with the exception of DQA1*0501 - DQB1*0301. This is also the most promiscuous of all HLA DQ. The same allele is found to bind a very high proportion of O-glycosylated peptides as compared to other HLA alleles (Figure 3C). While HLA DP alleles bind peptides with only a low portion of O-glycosylation, some of the HLA DR alleles (eg. DRB1*0401, DRB1*0701, or DRB1*0901) show mild tolerance to this posttranslational modification (Figure 3C). It is also of note that three out of the four HLA DQ alleles examined, seem to tolerate O-glycosylation somewhat more.

### Autoantigens

Autopathogenic T cells have been shown to play key roles in several autoimmune diseases such as multiple sclerosis, rheumatoid arthritis [39]. As the presence or absence of glycosylation is possibly associated with the efficacy of T cell recognition, it might also be involved in determining autoantigenic nature of a protein. To address this question, next we investigated entries in the Autoantigen Database [12]. According to our data autoantigens did not differ significantly in their amino acid composition from other components of the proteome. The same proportion of known autoantigens was identified to have a SigP (27.61%) as proteins of the human proteome.

The mean length of autoantigens was also similar to that of normal proteins (510 ± 23), although autoantigens with a SigP were found to be significantly longer (714 ± 51 amino acids) than the same type SwissProt entries (p < 0.001, Kolmogorov Smirnov test).

In this analysis, a protein was considered to be glycosylated if the glycosylation prediction suggested at least one probable glycosylation site within the molecule. A significantly higher proportion of autoantigens proved to be N-glycosylated (84.48%) than what was found when analyzing the members in the human proteome (73.54%) (p < 0.05, Kolmogorov Smirnov test). In contrast, no significant difference was observed between frequencies of O-glycosylation (61.2% as compared to 55.43%).

**Figure 4:**
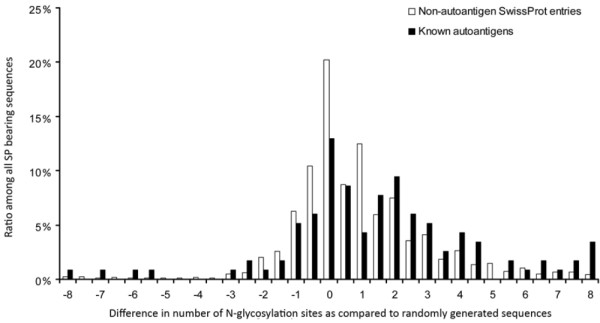
**Number of N-glycosylation sites in autoantigens and non-autoantigenic proteins**. Human SwissProt/UniProtKB proteins and known autoantigens (Autoantigen Database) with SigP were examined for the number of probable N-glycosylation sites. For each protein, random control sequences (n = 5) were generated consisting of the same amino acids and of the same length. The mean number of glycosylation sites found in random control sequences was subtracted from the number found in the given protein. The frequency of the difference values is shown for both the autoantigenic and the non-autoantigenic proteins.

To test if the observed difference in N-glycosylation of autoantigens was only due to the different protein length, we also assessed the glycosylation of length-matched randomly generated sequences as controls (n = 20 000). Autoantigens were found to contain significantly more N-glycosylation sites (+1.74 ± 0.32) than normal proteins (+0.91 ± 0.04) when both data sets were compared to randomly generated control sequences (Figure 4) (Kolmogorov Smirnov test, p < 0.001).

The mean number of probable glycosylation sites/protein was 2.48 ± 0.04 in the case of SigP- bearing non-autoantigenic SwissProt/UniProtKB entries, while it was 3.91 ± 0.35 among the autoantigenic sequences (mean ± standard error mean).

In order to confirm the above data, we also performed a Bayesian model-based analysis [37]. We calculated the posterior probability for each variable that it is directly dependent of a fixed target (outcome) variable Autoantigen (i.e., its probabilistic dependence is not mediated by other variables). This is formalized as being in the so called Markov Blanket Set of the variable Autoantigen, which is denoted with MBM (Autoantigen, Xi) (for an overview of this symmetric relation, see references in [36]). The posteriors are listed in Table 1 (variables with posteriors below 0.001 are not shown). This confirms that the length, number of N-glycosylation sites, number of disulfide bonds, number of transmembrane domains, number of strand domains are relevant variables to autoantigenicity.

**Table 1:** Correlation of structural features of proteins with their autoantigenic character

Investigated parameter	P (MBM (Autoantigen, Xi)|DN)	Kolmogorov-Smirnoff hypothesis test
Length	1	p < 0,001
EC N-glycosylation	1	p < 0,001
Total N-glycosylation	1	p < 0,001
Number of strand motifs	0.986774	p < 0,01
Number of transmembrane regions	1	p < 0,05
Number of turn motifs	0.005305	p < 0,05
Number of disulfide bridges	1	NS
Metal cofactor binding	0.097063	NS
Number of coiled coils	0.094628	NS
Number of alpha helices	0.014117	NS
Extracellular O-glycosylation	0.741702	NS
The presence of SigP	0.377562	NS
Intracellular N-glycosylation	0.132067	NS

Results of a Bayesian model-based analysis.

We calculated the posterior probability for each variable that it is directly dependent of the fixed variable, autoantigenicity of a protein (i.e., its probabilistic dependence is not mediated by other variables). This is formalized as being in the so called Markov Blanket Set of the variable Autoantigen, which is denoted with MBM (Autoantigen, Xi). Variables relevant to the autoantigen nature of proteins are listed in the table (posteriors below 0.001).

### Human-bacterial sequence matches

We analyzed 140000 bacterial SwissProt/UniProtKB entries found in the corresponding taxonomic division of the database to identify linear 100% sequence matches with the human proteome. The minimal peptide length was selected according to experimental data suggesting that most T cell epitopes are in the range of 8-22 amino acids. A short Python program was used for this function.

**Figure 5:**
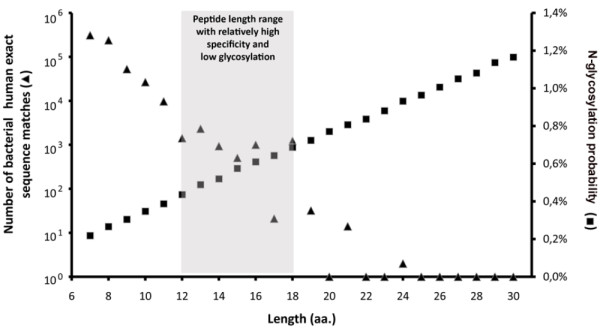
**Number of bacterial-human exact sequence matches**. Human SwissProt/UniProtKB proteins and known autoantigens (Autoantigen Database) containing SigP were scanned for short exact sequence matches with bacterial SwissProt/UniProtKB proteins. The number of these exact sequence matches exponentially decreases as their length grows. Black dots represent data obtained from the analysis of 140 337 bacterial proteins, while empty circles represent estimations based on analysis of 4000 randomly selected bacterial proteins. Data indicate that from ≥ 9 amino acid peptide lengths the number of bacterial-human sequence matches falls below 10^5^.

The number of continuous linear exact sequence matches between the whole bacterial and human proteome decreases logarithmically as the minimal length of the match is set higher (Figure 5). So does the number of proteins containing bacterial-human exact sequence matches. In the length range of immunologically relevant peptides (from the minimum of 8-9 aa for T cell epitopes), exact sequence matches between human and bacterial proteins are found in the order of 10^5^, which value is relatively low when compared to the order of magnitude of TCR diversity. Posttranslational protein modifications such as glycosylation may further decrease the probability of cross-reactive T cell epitope recognition.

Glycosylation of at least 9 aa long matches were examined the same way as the glycosylation of T cell epitopes. A linear matched sequence was considered glycosylated, if its amino acid position in the protein coincided with a probable glycosylation site. Glycosylated sequence matches were found to be slightly less N-glycosylated (4.05%) than O-glycosylated (5.39%) (Figure 2, grey columns). This difference was higher if only autoantigens were examined. Human-bacterial sequence matches derived from these proteins were predicted to be significantly (p < 0.001) less N-glycosylated (3.45%) than O-glycosylated (6.89%). In contrast to the human-bacterial sequence matches, the N- and O-glycosylation of autoantigen and non-autoantigen protein-derived sequence matches did not differ significantly.

**Figure 6:**
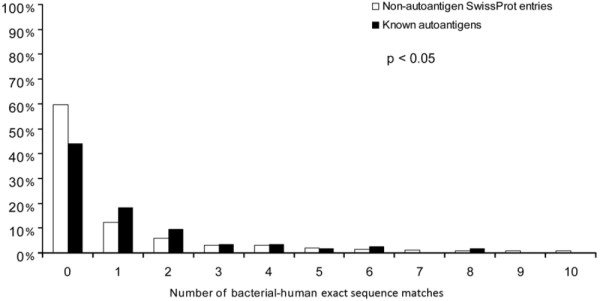
**Autoantigenic sequences with bacterial-human sequence matches**. The frequency of proteins that contain a given number of bacterial-human exact sequence matches (min. 9 aa) is shown for autoantigens and non-autoantigenic SwissProt entries. Significantly more autoantigens have at least one bacterial-human exact sequence match than non-autoantigenic human SwissProt entries. Furthermore, autoantigens also bear significantly more matches as compared to non-autoantigenic ones (p < 0.05, Kolmogorov-Smirnov).

A larger proportion of known autoantigens (56.04%) contained at least 9 aa long human-bacterial matches compared to non-autoantigenic human SwissProt/UniProtKB entries (40.40%) (Figure 6, p < 0.05, Kolmogorov Smirnov test).

To confirm that the reduced glycosylation of bacterial-human sequence matches was not due to the short length of the corresponding peptides, we tested the glycosylation probabilities along the full length of proteins carrying sequence matches. The mean probability of N-glycosylation in the region of sequence matches was below the 50% "threshold" of the prediction (43.92%).

On the contrary, when O-glycosylation probability was tested, the matching sequences of bacterial-human proteins had glycosylation probability above the threshold value (56.80%). Both N- and C-terminally to the matched sequences, the mean O-glycosylation probability was above the glycosylation threshold (50%), while the mean probability varied in regions farther from the exact sequence match.

## Discussion

Several autoimmune disease models have been shown to be dependent on posttranslational modifications of relevant Ags (e.g. on acetylation of a myelin basic protein peptide (MBP-Ac1-11) [40] or on the isoaspartyl-modification of a self Ag in a mouse model of systemic lupus erythematosus [41] or of peptides in DR4-transgenic mice expressing human type II collagen [42].

Surprisingly, the role of the most common PTM, glycosylation, is poorly understood not only in the development of autoimmune diseases, but also in basic immune processes like T cell recognition.

Instead of using glycosylation-related annotations of SwissProt/UniProtKB or one of the PTM- specific databases (dbPTM or O-GLCBASE 6.0 [43,44]), in our work, we chose to use Artificial Neural Network-based glycosylation prediction tools. By this approach, we could avoid errors of comparison resulting from publication bias, the lack of negative results and the diverse methods used to characterize glycosylation of molecules. Beside the numerous advantages, the authors are also aware of disadvantages of *in silico *technologies. Prediction methods use mainly stochastic algorithms, and a known portion of all provided answers is incorrect. This error, however, can be taken into account precisely, and be corrected during statistical analysis, while it is very hard to assess the systemic bias caused by errors in database entries.

In our study, we compared the glycosylation of experimentally verified T cell epitopes of the IEDB to that of the human proteome. According to our present knowledge, the most common types of glycosylation, N- and O-glycosylation, occur only in the rER. Nevertheless, earlier studies on glycosylation of the proteome have not yet considered the presence or absence of a (SigP) [45]. Our data are in accordance with the results of former studies regarding the frequency of glycosylation. Earlier, based on the analysis of a smaller pool of 6000 human proteins, it has been demonstrated that N-glycosylation preferentially occurs on proteins associated with transport and binding functions [45]. In another article, the clustering phenomenon of O-glycosylation was also reported for 221 glycoproteins [15]. Furthermore, N-glycosylation was reported to occur preferentially in the central and N-terminal regions of proteins, whereas O-glycosylation showed preference for both the N- and C-termini [46].

In this study, we found very low glycosylation of the experimentally verified T cell epitopes in comparison with the glycosylation of the entire human proteome. This is logical given the necessity of a TCR to interact simultaneously with the alpha helices of the MHC molecule and the presented peptide. If this peptide is glycosylated, the trimolecular complex formation might be prevented because of steric reasons. Such interference of epitope glycosylation with TCR binding has been indeed, reported already [1-3]. However, on the other hand, there are also data on recognition of glycopeptide epitopes by T cells [2,47-51].

Epitope glycosylation may not only interfere with TCR binding but also with binding of a peptide to MHC. Many of the T cell epitopes in the IEDB may represent immunodominant epitopes, those few peptides that are found in the majority of peptide-MHC complexes during an immune response after natural intracellular processing of an Ag [52,53]. Whether glycopeptides suffer any disadvantage because of their glycan moiety when competing for MHC binding, appears to be an interesting question with practical consequences.

The fact that we found only a minimal proportion of MHC II-restricted peptides to be glycosylated in case of most MHC alleles, supports the notion that the glycan moiety might indeed negatively affect glycopeptide presentation. On the other hand, the relatively high proportion of glycosylated peptides predicted to bind to certain HLA alleles, might be an artifact caused by the highest sensitivity, thus, a bit lower specificity of the prediction. Higher permissivity of these alleles towards probable N- but not O-glycosylation of peptides, however, might have relevance to pathological immune responses, but this difference still needs to be experimentally confirmed.

T cell epitope glycosylation may also have implications to autoimmunity. It may be hypothesized that within the thymus, glycosylation of peptides interferes with their presentation by protecting proteolytic cleavage sites. This would be a mechanism parallel to that already reported in case of O-glycosylation [3]. Not only the fact of glycosylation, but also the size and complexity of the N-glycan moiety might play a significant role in the presentation of N-glycosylated peptides [4]. In the absence of presentation, certain glycosylated parts of proteins may indeed, escape thymic central tolerance induction. In the periphery, however, glycosidase enzymes (of microbial or inflammatory cell origin) may attack the glycan moieties, and the naked peptides may become target neoepitopes of an autoimmune recognition. In line with this concept, our earlier data showed that elevated glycosidase activities were indeed, predictors of rheumatoid arthritis [54]. Proteins with higher number of N-glycosylation sites thus, are more probably recognized by autoimmune T cells than other proteins. In this work, we observed that the N-glycosylation of autoantigens was significantly higher than that of length-matched randomly generated sequences or normal proteins.

Molecular mimicry has been shown in several human autoimmune diseases [55] including multiple sclerosis [56], Sydenham's chorea [57] rheumatic heart disease [58], autoimmune thyroiditis [59], Chagas heart disease [60], systemic lupus erythematosus, etc. [61]. Since overlapping human-bacterial sequences may trigger autoimmune processes, their immune recognition might be substantial regarding autoimmunity. We found a reduced glycosylation of the linear human-bacterial exact sequence matches. As both T cell epitopes and bacterial-human exact sequence matches are much shorter than full length proteins, the reduced glycosylation observed in our work, could have been attributed to the difference in peptide length. Indeed, we found that glycosylation probability increases in parallel with the peptide length. However, we have shown in this study that the reduced glycosylation of neither the T cell epitopes, nor the human-bacterial exact sequence matches were the function of peptide length only.

According to most *in vitro *results, low glycosylation rates of human T cell epitopes may be a prerequisite for an effective immune recognition. Given the necessity of both hypoglycosylation and the highest specificity for an epitope sequence; the length of experimentally verified human T cell epitopes occupies a niche optimized for these two features (Figure 6). At the relevant peptide lengths for MHC-II-restricted T cell epitopes, only a relatively low number of bacterial-human exact sequence matches can be found, and simultaneously the probability of peptide glycosylation is also minimal.

## Conclusion

There is a high need to improve the efficacy of T cell epitope prediction [62]. Since currently available methods ignore epitope glycosylation, taking it into account, might yield in a more precise T cell epitope identification. Until glycopeptides will be available on a large scale for cellular immunology studies, this work may enable us to gain the first system biology insight into the largely unknown role of antigen glycosylation in T cell immunity. In spite of its limitations, it may provide important clues for the design of future experimental work.

## Abbreviations

Ag: antigen; AUC: area under the curve; IEDB: ImmunEpitope Database; MBM: Markov blanket model; MBP: myelin basic protein; MHC: major histocompatibility complex; MCMC: Markov chain Monte Carlo; MUC1: Mucin-1 protein; NXS/T: an amino acid triplet, where N stands for asparagine, X for any amino acid except for proline and the third amino acid is either serine or threonine; PTM: posttranslational modification; SigP: signal peptide; TAP: transporter of antigen processing.

## Competing interests

The authors declare that they have no competing interests.

## Authors' contributions

Data acquisition and analysis has been performed by TGSz, RP and IT. Bayesian analysis was carried out by AP. Intellectual contributions to the manuscript were provided by AF, OL, TGSz, LT, GN and EIB. All authors read and approved the final manuscript
